# The General Practitioner and the Consultant

**Published:** 1907-04-20

**Authors:** 


### The General Practitioner and the Consultant.

For the last 600 years the profession of medicine in London and the larger towns in England has never been without two classes of practitioner. One class, and always the more numerous, has attended to the lesser ailments of the people; the other has been summoned, on account of its mature experience, to difficult and doubtful cases. Consultations were compulsory as long as the Barbers' Company and the United Company of the Barbers and Surgeons controlled the profession. It was enacted from very early days that if any member of the company failed to present a patient under his care who was seriously ill, or who was in danger of losing a limb, he was fined or reprimanded. The presentation was made to the company, and some member, generally the master or one of the wardens, was detailed to consult and report. The rule was admirable in its inception, but it worked badly in practice as soon as the right of consultation became a perquisite of the superior officers, since they were not always the men best fitted to give an opinion. In the seventeenth and eighteenth centuries the physicians arrogated to themselves a remarkable position, for they treated patients by "bills" or prescriptions, sometimes given on the mere report of an apothecary as to the condition of a patient whom they had never seen.

The establishment of hospitals, or rather the foundation of medical schools in connection with hospitals, gave rise to a special class of practitioner who was the forerunner of the present consultant. His hospital experience gave him opportunities of seeing a very large number of cases, whilst students, taking him as their master, called him in to consultation when they had themselves attained to practice. This is still the usual way to consultant rank, but the more perfect method is still followed in many parts of the country, sometimes in medicine, more often in midwifery, seldom in surgery. A practitioner eminent among his fellows by skill, experience, or general ability, is referred to in many cases of doubt and difficulty. Such a man has already been through the troubles of those who bring their patients to him, and he can therefore sympathise with them the more readily, and can act in the most friendly spirit of co-operation.

With surgeons and those who practise the special departments of the eye, ear, and throat, the qualifications which are useful in medicine and midwifery must be supplemented either by natural or acquired skilfulness of hand, a skilfulness which can only be attained by long and constant practice. Dr. Joachim, the prince of violinists, used to say that if he neglected to practise for a few days he knew it himself; if he neglected to practise for a few weeks the public knew it. So, too, a surgeon must be operating daily if he is to perform a gastro-enterostomy or an excision of the larynx with the speed and certainty which alone lead to success. Such skill and practice are attained by those attached to hospitals; but in addition to these qualifications, others are essential to make a trustworthy consultant. He must be absolutely straightforward in his dealings with the patient, and frankly loyal to the medical practitioner who confides in him. There is no question of breach of faith with the best men who have already attained the first rank, for they would not have gained their position unless they had been beyond reproach.

But the desire to have a code of rules drawn up to govern the ethics of consultation shows that some consultants fail in their duty to the profession, just as it is certain that some general practitioners fail in their duties to consultants either intentionally or by the discourtesy of neglect. In this code it is suggested that the general practitioner should nominate the consultant, and although this may be serviceable as a general rule, it is often impolitic to press it. There are often many reasons which lead a patient to express a decided preference for one consultant rather than another, and so long as this preference is exercised with discretion it should not be overridden. The chief operating surgeons approach as nearly as possible to the position of pure consultants. They rarely see a patient, except on the recommendation of a general practitioner, and never operate unless the usual medical attendant has been given the opportunity of attending. In the few cases where a patient comes without a personal introduction it is usual to communicate with the practitioner, for when a patient is at first averse to any communication a few words of explanation always end in his acquiescence. Too often, however, a polite letter of explanation to the practitioner may be ignored in such cases, although the true consultant will invariably continue this practice.

